# Sex-based differences in antibody-mediated rejection among highly sensitized kidney transplant recipients

**DOI:** 10.1093/ckj/sfag367

**Published:** 2026-10-19

**Authors:** Evelyn Hermida, Carolt Arana, Jordi Rovira, Edoardo Melilli, Francesc Moreso, Marta Crespo, Carme Facundo, Anna Vila-Santandreu, Maria José Perez-Saez, Alexandre Favà, Cinthia Baldallo, Cristina Canal, Javier Paúl-Martinez, Jordi Comas, Jaume Tort, Eduard Palou, Fritz Diekmann, Pedro Ventura-Aguiar

**Affiliations:** Department of Nephrology and Kidney Transplantation, Institut Clínic de Nefrologia i Urologia (ICNU), Hospital Clínic de Barcelona, Barcelona, Spain; Laboratori Experimental de Nefrologia i Trasplantament (LENIT), Institut d’Investigacions Biomèdiques August Pi i Sunyer (IDIBAPS), Barcelona, Spain; Facultat de Medicina i Ciències de la Salut, Universitat de Barcelona (UB), Barcelona, Spain; Department of Nephrology and Kidney Transplantation, Institut Clínic de Nefrologia i Urologia (ICNU), Hospital Clínic de Barcelona, Barcelona, Spain; Laboratori Experimental de Nefrologia i Trasplantament (LENIT), Institut d’Investigacions Biomèdiques August Pi i Sunyer (IDIBAPS), Barcelona, Spain; Facultat de Medicina i Ciències de la Salut, Universitat de Barcelona (UB), Barcelona, Spain; RICORS 2040 RD 21/0005/0022, Barcelona, Spain; Laboratori Experimental de Nefrologia i Trasplantament (LENIT), Institut d’Investigacions Biomèdiques August Pi i Sunyer (IDIBAPS), Barcelona, Spain; RICORS 2040 RD 21/0005/0022, Barcelona, Spain; Nephrology Department, Bellvitge University Hospital, Barcelona, Spain; Nephrology Department, Hospital Universitari Vall d’Hebron, Barcelona, Spain; Nephrology Department, Hospital del Mar Research Institute, Hospital del Mar, Barcelona, Spain; Nephrology Department, Fundació Puigvert, Barcelona, Spain; Nephrology Department, Hospital German Trias i Pujol, Barcelona, Spain; Nephrology Department, Hospital del Mar Research Institute, Hospital del Mar, Barcelona, Spain; Nephrology Department, Bellvitge University Hospital, Barcelona, Spain; Nephrology Department, Hospital Universitari Vall d’Hebron, Barcelona, Spain; Nephrology Department, Fundació Puigvert, Barcelona, Spain; Nephrology Department, Hospital German Trias i Pujol, Barcelona, Spain; Organització Catalana de Trasplantaments (OCATT), Barcelona, Spain; Organització Catalana de Trasplantaments (OCATT), Barcelona, Spain; Immunology Department, Hospital Clínic de Barcelona, Barcelona, Spain; Department of Nephrology and Kidney Transplantation, Institut Clínic de Nefrologia i Urologia (ICNU), Hospital Clínic de Barcelona, Barcelona, Spain; Laboratori Experimental de Nefrologia i Trasplantament (LENIT), Institut d’Investigacions Biomèdiques August Pi i Sunyer (IDIBAPS), Barcelona, Spain; RICORS 2040 RD 21/0005/0022, Barcelona, Spain; Department of Nephrology and Kidney Transplantation, Institut Clínic de Nefrologia i Urologia (ICNU), Hospital Clínic de Barcelona, Barcelona, Spain; Laboratori Experimental de Nefrologia i Trasplantament (LENIT), Institut d’Investigacions Biomèdiques August Pi i Sunyer (IDIBAPS), Barcelona, Spain; RICORS 2040 RD 21/0005/0022, Barcelona, Spain

**Keywords:** antibody-mediated rejection, donor-specific antibodies, highly sensitized recipients, HLA-DQ eplet mismatch, sex differences

## Abstract

**Background:**

Antibody-mediated rejection (AMR) remains a major determinant of graft failure in kidney transplantation, particularly among highly sensitized recipients. Whether biological sex independently influences the incidence and determinants of AMR in this high-risk population remains uncertain. We hypothesized that recipient sex may be associated with early AMR risk beyond conventional immunologic risk factors. We therefore evaluated the association between sex and early AMR, explored sex-specific immunologic determinants, and assessed the impact of AMR on long-term graft survival.

**Methods:**

We performed a multicenter retrospective cohort study of 438 highly sensitized deceased-donor kidney transplant recipients with calculated panel reactive antibody ≥90% transplanted in Catalonia, Spain, between 2015 and 2021. The primary outcome was AMR within the first post-transplant year. Secondary outcomes included 5-year death-censored graft survival and sex-specific associations with AMR. Median follow-up was 42.5 months.

**Results:**

Within the first year, 51 recipients (11.6%) developed AMR. Men exhibited a higher incidence of AMR compared with women (16.2% vs. 8.5%; *P* = .01). In sex-stratified analyses, antibody strength was the dominant determinant in men, whereas human leukocyte antigen DQ beta 1 (HLA-DQB1) eplet mismatch was more strongly associated with AMR in women, although formal interaction testing was not statistically significant. AMR occurring within the first year was the strongest predictor of 5-year graft failure (hazard ratio 8.3; *P* < .001), while recipient sex was not independently associated with graft loss.

**Conclusions:**

In highly sensitized recipients, male sex was modestly associated with early rejection. AMR emerged as the main driver of long-term graft loss. The impact of epitope-level HLA mismatch on AMR risk may differ between sexes, particularly at the HLA-DQB1 locus, and warrants further investigation.

## Graphical Abstract

**Graphical Abstract:**
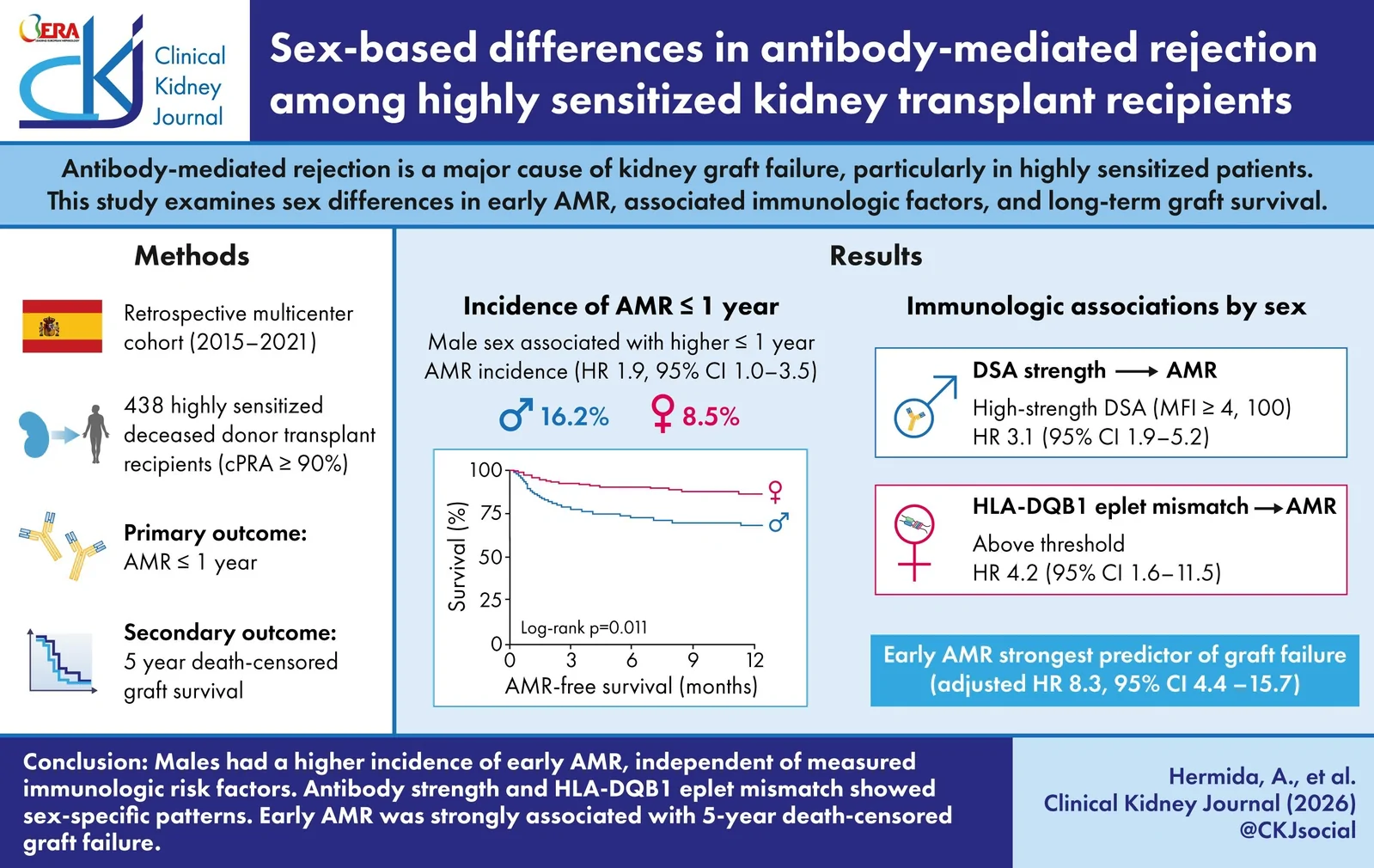

KEY LEARNING POINTS **What was known:**

- Antibody-mediated rejection (AMR) is a major cause of kidney allograft failure, particularly among highly sensitized recipients.
- Although sex-related differences in sensitization are well recognized, their influence on post-transplant AMR risk remains uncertain.
- The relative contribution of antibody burden and molecular human leukocyte antigen (HLA) compatibility to AMR risk according to sex has not been extensively studied.

**This study adds:**

- In this multicenter cohort of highly sensitized kidney transplant recipients, men showed a higher incidence of AMR during the first post-transplant year.
- Antibody strength and HLA-DQB1 eplet mismatch were associated with AMR and showed different patterns of association in exploratory sex-stratified analyses.
- Early AMR was consistently associated with poorer long-term graft survival.

**Potential impact:**

- These findings reinforce the importance of a multidimensional assessment of immunologic risk in highly sensitized kidney transplant recipients.
- Donor-specific antibody strength and molecular HLA compatibility may provide complementary information for AMR risk assessment.
- Larger prospective studies are needed to determine whether the observed sex-related associations have implications for risk stratification and post-transplant management.

## INTRODUCTION

Antibody-mediated rejection (AMR) represents a major cause of long-term allograft failure despite advances in immunosuppression [1, 2]. Recipients who develop AMR within the first year post-transplant experience substantially worse long-term outcomes, highlighting the need for improved risk assessment and early identification of patients at high risk [2, 3].

Pre-existing sensitization is a central determinant of AMR. Anti-human leukocyte antigen (HLA) antibodies arise following exposure to non-self-antigens, most commonly through prior transplantation, blood transfusion, or pregnancy [4, 5]. Consequently, highly sensitized recipients represent one of the most immunologically complex populations in contemporary transplantation [6−8].

Traditional measures of immunologic risk, including calculated panel reactive antibody (cPRA), the presence and strength of donor-specific antibodies (DSAs), and antigen-level HLA mismatch, provide important but incomplete information. Molecular HLA mismatch at the eplet level has improved the characterization of alloimmune risk beyond conventional antigen matching [8, 9]. However, these established immunological markers do not fully explain the marked heterogeneity in post-transplant outcomes among patient subgroups.

Biological sex has increasingly been recognized as an important modifier of immune responses [10−12]. Across infectious, autoimmune, and vaccine settings, women generally mount more robust humoral responses than men, a phenomenon attributed to hormonal regulation, X-linked immune regulatory genes, and sex-specific differences in antibody fragment crystallizable (Fc) glycosylation and effector function [10, 13].

In transplantation, sex-based differences have been described in access to transplantation, sensitization patterns, and cumulative immunologic exposure [14]. However, the independent impact of biological sex on rejection and long-term graft survival remains uncertain. Large registry analyses have reported inconsistent associations, with sex effects frequently attenuating after adjustment for immunologic risk factors. Notably, most prior investigations have evaluated broad transplant populations, in whom alloimmune burden is heterogeneous and often modest [15, 16]. Whether biological sex independently modifies the incidence or determinants of AMR among highly sensitized recipients, those at the extreme end of alloimmune risk (cPRA ≥ 90%), has not been specifically addressed [17].

To address these gaps, we conducted a multicenter cohort study of highly sensitized (cPRA ≥ 90%) deceased donor kidney transplant recipients to determine whether biological sex independently affects the incidence of 1-year AMR; to assess whether the determinants of AMR differ between men and women; and to evaluate whether sex modifies the long-term prognostic impact of early AMR on 5-year death-censored graft survival.

## MATERIALS AND METHODS

### Study population and follow-up

We conducted a multicenter, observational, retrospective study including highly sensitized adult recipients who underwent deceased-donor kidney transplantation between July 2015 and December 2021 in six hospitals in Catalonia, Spain. Median follow-up time was 42.5 months [interquartile range (IQR) 23–58]. Recipients were considered highly sensitized if their cPRA class I + II was ≥90% on the day of transplantation. The study was conducted in accordance with the principles of the Helsinki and Istanbul Declarations, as well as national and local laws and standards of good clinical practice (IRB reference: HCB/2022/0692).

Patients were transplanted within the framework of regional allocation programs for highly sensitized candidates in Catalonia, including the HIPERCAT program for recipients with cPRA 90%–98% and the national PATHI program for recipients with cPRA ≥98%. During the study period, 470 kidney transplants were performed across the participating centers. Because the study focused on the impact of sex on acute rejection and graft outcomes, we excluded recipients with primary graft failure (*n* = 18), defined as absent graft perfusion or transplantectomy within 48 h after transplantation, and recipients lost to follow-up within the first 30 days (*n* = 14).

Clinical and immunologic data were collected from electronic medical records. Delayed graft function was defined as the need for dialysis within the first post-transplant week. Graft failure was defined as return to dialysis or retransplantation. Death with a functioning graft was censored for the analysis of death-censored graft survival. AMR-free survival was defined as the time to the first AMR episode. Patients were censored at the time of the event or at last follow-up through 31 December 2023.

### Immunosuppression

Induction immunosuppression was primarily based on thymoglobulin, administered according to center-specific protocols at 1–1.5 mg/kg per dose for 4–6 doses, adjusted based on hematologic parameters. In a subset of recipients, the cPRA ≥90% result from the day of transplantation became available only retrospectively; these patients received basiliximab induction (20 mg on day 0 and day +4) instead of lymphocyte-depleting therapy.

Maintenance therapy included corticosteroids, tacrolimus with initial target trough levels 10–12 ng/ml, and either mycophenolic acid (720 mg twice daily) or a mTOR inhibitor with initial target trough levels of 4 ng/ml.

In patients with preformed DSAs (pfDSAs) with a mean fluorescence intensity (MFI) ≥3000 and a negative flow cytometry crossmatch (FCXM), rituximab (375 mg/m^2^) was added to induction therapy. In recipients with a positive FCXM, intravenous immunoglobulin (1–2 g/kg) and plasma exchange (5–7 sessions of one plasma-volume exchange) were also used.

### Immunological assessments

Single-antigen bead assays were performed using Lifecodes LSA Class I and II on a Luminex 200 IS platform, with results processed using Match IT Antibody software. Antibodies against HLA-DP were excluded because of incomplete availability and characterization during the study period. All transplants were performed after a negative complement-dependent cytotoxicity crossmatch, performed using total donor lymphocytes without separate T- and B-cell assessment. From 2017 onward, FCXM formed part of the routine pre-transplant immunological assessment. Auto-FCXM was not routinely performed and was reserved for selected cases of otherwise unexplained FCXM positivity in which autoantibody-mediated reactivity was suspected. FCXM results were recorded as the final clinical interpretation (positive or negative). Information regarding T-cell and B-cell FCXM reactivity and other assay-level technical parameters was not systematically available in the multicenter database and therefore could not be evaluated separately.

### HLA typing and eplet mismatch

Human leukocyte antigen (HLA) typing was performed using PCR-SSP for donors and PCR-SSO for recipients between 2015 and 2018, covering HLA-A, -B, -C, -DR, and -DQ loci. High-resolution HLA typing was available for 136 donors (31.1%) and 84 recipients (19.2%). Among recipients with high-resolution typing, HLA-DQA1 was directly available in 37 cases. When high-resolution typing was unavailable, the most common haplotypes were imputed based on Spanish population frequencies [18]. Donor–recipient compatibility was evaluated using HLAMatchmaker 3.16.7, and eplet mismatches for class I and class II loci were calculated using HLAMatchmaker algorithms. The primary analyses were based on the total HLA-DQ beta 1 (HLA-DQB1) eplet mismatch score (all HLA-DQB1).

### Diagnosis of allograft rejection

Rejection episodes were classified according to the original pathology reports issued at the time of biopsy, using the Banff classification applicable in routine clinical practice at the time [19].

Biopsies were performed either for-cause or per protocol, according to center criteria. Protocol biopsies were performed at approximately 1 week, 3 months, and/or 12 months post-transplant. Borderline rejection episodes were not classified as T cell-mediated rejection (TCMR).

### Statistical analysis

Continuous variables were presented as mean ± standard deviation (SD) or median (IQR), and categorical variables as counts (percentages). Receiver operating characteristic (ROC) curves assessed the ability of age, MFI, and eplet mismatch to predict AMR; optimal cut-offs were derived by the Youden index. Group comparisons used parametric or nonparametric tests. Cox proportional hazards models evaluated factors related to AMR within 1 year and graft failure within 5 years. As a sensitivity analysis, stabilized inverse probability of treatment weighting (IPTW) based on propensity scores for male recipient sex was performed using age, previous kidney transplantation, cPRA, previous transfusion, pfDSA, and HLA-DQB1 eplet mismatch. Balance was assessed using absolute standardized mean differences (SMDs; <0.10 considered adequate), and 1-year AMR was evaluated using an IPTW-weighted Cox model. Variables with *P* < .20 in univariate analyses were entered into the multivariable models, together with clinically relevant covariates selected a priori. Hazard ratios (HRs) with 95% confidence intervals (CIs) were reported. Kaplan–Meier methods and log-rank tests were used for survival analyses. Statistical significance was defined as *P* < .05. All analyses were performed using SPSS version 29.0 (IBM Corporation, Armonk, NY, USA). Missing data were minimal for variables included in multivariable analyses. Information on prior pregnancies was incomplete and therefore analyzed descriptively and not included in adjusted models. Multivariable models for death-censored graft survival were designed to evaluate baseline predictors of graft failure; accordingly, post-transplant events such as major infections were not included as conventional baseline adjustment covariates.

## RESULTS

### Cohort characteristics

The cohort included 438 highly sensitized kidney transplant recipients, of whom 179 (41%) were men and 259 (59%) were women (Fig. 1).

**Figure 1:**
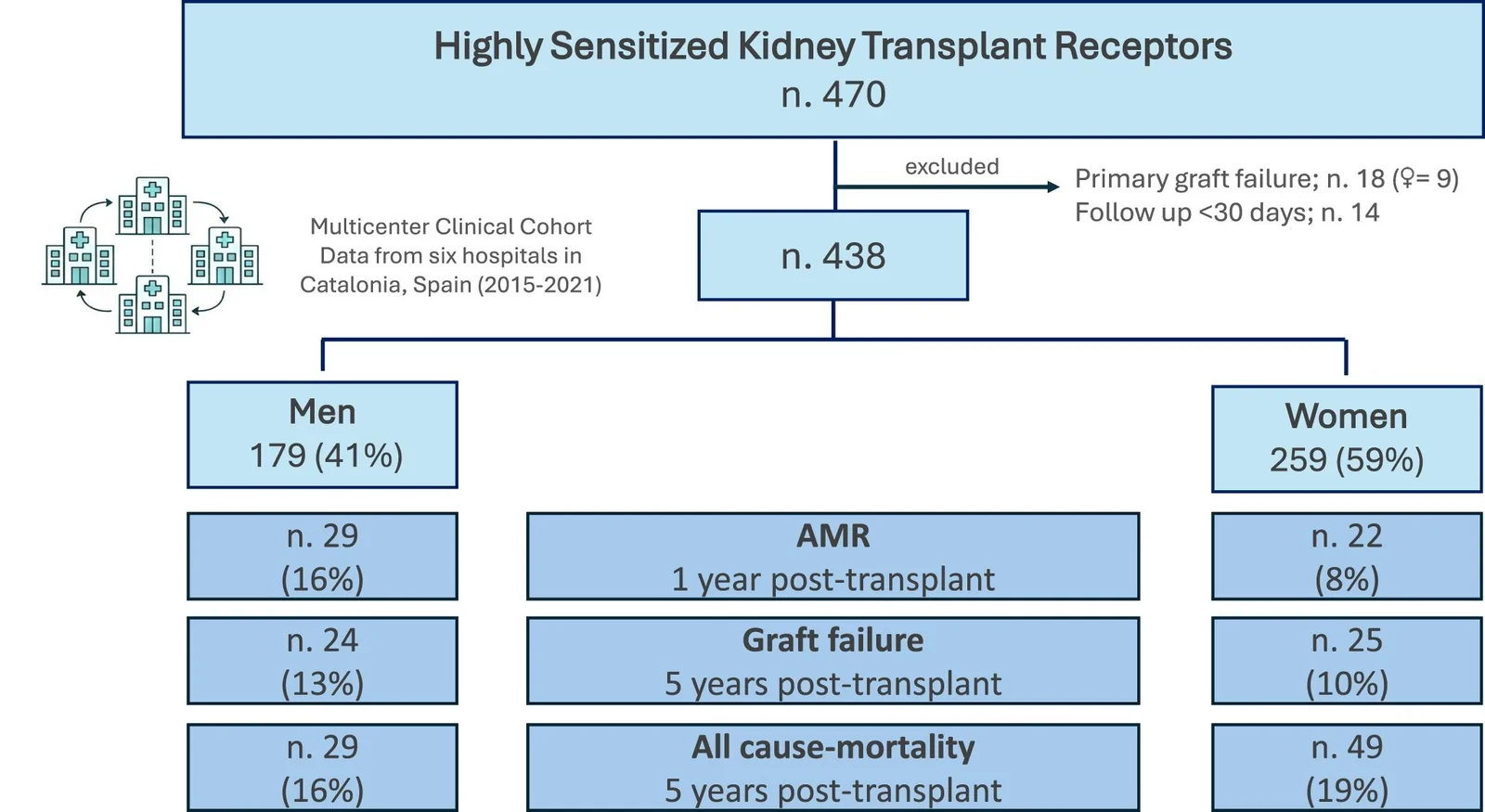
Study flow diagram and outcomes by sex in highly sensitized kidney transplant recipients. A total of 470 patients were initially included. After excluding 32 patients (primary graft failure, *n* = 18 [including 9 women], and follow-up <30 days, *n* = 14), the final cohort comprised 438 recipients, including 179 men (41%) and 259 women (59%). At 1-year post-transplant, AMR occurred in 29 men (16%) and 22 women (8%). At 5 years, graft failure was observed in 24 men (13%) and 25 women (10%). All-cause mortality at 5 years was 29 (16%) in men and 49 (19%) in women. Percentages are calculated within each sex.

Recipient age (mean 55 years), hypertension, and diabetes prevalence did not differ significantly between sexes (Table 1). Men were more likely to have undergone previous kidney transplantation (91% vs. 57%; *P* < .001), whereas 83% of women had prior pregnancies. Pretransplant blood transfusions did not differ between sexes. Induction and maintenance immunosuppression were comparable.

**Table 1:** Baseline recipient, donor, and immunologic characteristics by sex and AMR status.

	Overall 438 (100%)	Men 179 (40.9%)			Women 259 (59.1%)			Men vs. Women (overall)
		No AMR (*n* = 150)	AMR (*n* = 29)	*P*	No AMR (*n* = 237)	AMR (*n* = 22)	*P*	*P*
Recipient characteristics								
Age (years), mean (SD)	55 (12)	55 (12)	50 (13)	.05	56 (12)	52 (13)	.19	.17
Age < 50 years, *n* (%)	143 (33)	50 (33)	17 (59)	.01	64 (27)	12 (55)	.01	.76
Hypertension, *n* (%)	414 (95)	142 (96)	25 (93)	.32	226 (97)	21 (96)	.69	.62
Diabetes, *n* (%)	110 (26)	34 (23)	5 (19)	.06	66 (28)	5 (23)	.56	.20
Previous KT, *n* (%)	311 (71)	136 (90)	27 (93)	.67	137 (58)	11 (50)	.48	<.001
*#* Previous KT, median (IQR)	1 (1–2)	1 (1–1)	1 (1–2)	.24	1 (1–2)	2 (1–2)	.04	.69
Pregnancies before KT, *n* (%)^a^	161 (83)				147 (82)	14 (93)	.27	
Transfusions before KT, *n* (%)	201 (46)	68 (45)	14 (48)	.88	113 (48)	6 (27)	.07	.48
Donor characteristics								
Age (years), mean (SD)	58 (12)	57 (12)	55 (9)	.50	58 (13)	60 (10)	.56	.70
Women, *n* (%)	164 (38)	55 (34)	9 (35)	.80	87 (37)	13 (59)	.04	.63
Brain death, *n* (%)	284 (65)	92 (61)	15 (52)	.33	164 (69)	13 (59)	.33	.07
Ischemia time (h), mean (SD)	15 (7)	15 (6)	13 (7)	.06	15 (7)	16 (7)	.81	.85
eGFR (ml/min/1.73 m^2^), median (IQR)	96 (79–107)	98 (83–110)	98 (97–100)	.85	95 (74–105)	93 (77–99)	.25	.54
Induction therapy				.81			.74	.20
Anti-thymocyte globulin (ATG), *n* (%)	404 (92)	136 (91)	27 (93)		221 (94)	21 (96)		
Basiliximab, *n* (%)	34 (8)	14 (9)	2 (7)		16 (6)	1 (5)		
Maintenance therapy				.81			.34	.09
TAC/MPA/prednisone TAC/mTORi/prednisone Others	352 (80) 65 (15) 21 (5)	121 (81) 26 (17) 3 (2)	24 (83) 4 (14) 1 (3)		192 (81) 30 (13) 15 (6)	15 (68) 5 (23) 2 (9)		
Delayed graft function	128 (29)	47 (31)	13 (45)	.16	63 (27)	5 (23)	.67	.18
Immunologic characteristics at transplant								
cPRA (%), median (IQR)	98 (95–99)	98 (95–100)	99 (97–100)	.11	97 (95–99)	98 (96–99)	.38	.25
cPRA > 98%, *n* (%)	187 (43)	66 (44)	18 (62)	.07	93 (39)	10 (46)	.57	.14
FCXM (positive), *n* (%)	24 (5)	6 (5)	6 (24)	<.001	12 (6)	0 (0)	.26	.31
Class I in Luminex (positive), *n* (%)	330 (75)	110 (73)	26 (90)	.06	176 (74)	18 (82)	.43	.80
Class II in Luminex (positive), *n* (%)	366 (84)	125 (83)	25 (86)	.70	194 (82)	22 (100)	.03	.91
pfDSA at day of transplant, *n* (%)	116 (27)	30 (20)	19 (66)	<.001	59 (25)	8 (36)	.24	.73
pfDSA *n*, median (IQR)	1 (1–2)	1 (1–2)	2 (1–2)	.02	1 (1–1)	1 (1–1)	.25	.21
pfDSA MFI, median (IQR)	2982 (1401–6740)	2366 (1251–5052)	6020 (2345–10 370)	.07	2900 (1432–5043)	3991 (1698–18 362)	.34	.48
pfDSA MFI, ≥3000, *n* (%)	56 (48)	11 (38)	13 (68)	.04	28 (49)	4 (50)	.96	.75
pfDSA MFI, ≥4100, *n* (%)	45 (40)	8 (27)	13 (68)	.01	20 (34)	4 (50)	.41	.69
Class I pfDSA positive, *n* (%)	37 (8)	12 (8)	4 (14)	.32	18 (8)	3 (14)	.32	.76
Class II pfDSA positive, *n* (%)	59 (14)	14 (9)	7 (24)	.02	32 (14)	6 (27)	.08	.38
pfDSA DR positive, *n* (%)	23 (5)	8 (5)	3 (10)	.30	11 (5)	1 (5)	.98	.49
pfDSA DQ positive, *n* (%)	25 (6)	4 (3)	2 (7)	.25	15 (6)	4 (18.2)	.04	.08
HLA-DRB1 *#* MM, median (IQR)	1 (0–1)	1 (0–1)	1 (1–1)	.22	1 (0–1)	1 (1–1)	.45	.09
HLA-DQB1 *#* MM, median (IQR)	1 (0–1)	0 (0–1)	1 (0–1)	.31	0 (0–1)	1 (0–1)	.03	.30
HLA-DQA1 *#* MM, median (IQR)	0 (0–1)	0 (0–1)	0 (0–1)	.24	0 (0–1)	1 (0–1)	.10	.32
All HLA Class II eplet MM (0–50), median (IQR)	13 (3–21)	13 (3–21)	17 (8–21)	.22	12 (1–21)	20 (9–24)	.04	.07
Verified HLA Class II eplet MM (0–19), median (IQR)	5 (1–8)	5 (1–8)	7 (3–8)	.10	4 (0–8)	8 (4–9)	.02	.17
All HLA-DRB1 eplet MM (>7), *n* (%)	191 (44)	67 (45)	13 (45)	.98	103 (44)	10 (46)	.85	.83
All HLA-DQB1 eplet MM (>4), *n* (%)	216 (49)	76 (51)	17 (59)	.43	106 (45)	17 (77)	.003	.36
All HLA-DQA1 eplet MM (>1), *n* (%)	176 (40)	53 (35)	13 (45)	.33	97 (41)	13 (59)	.09	.24
Verified HLA-DRB1 eplet MM >2 (vs. 0–2), *n* (%)	196 (45)	70 (47)	13 (45)	.86	101 (43)	12 (55)	.28	.57
Verified HLA-DQB1 eplet MM >2 (vs. 0–2), *n* (%)	163 (37)	58 (39)	15 (52)	.19	74 (31)	16 (73)	<.001	.20
Verified HLA-DQA1 eplet MM >1 (vs. 0–1), *n* (%)	176 (40)	36 (24)	12 (41)	.05	72 (30)	10 (46)	.15	.24

^a^Data on prior pregnancies were available for 75% of women.

Abbreviations: AMR, antibody-mediated rejection; ATG, anti-thymocyte globulin; cPRA, calculated panel reactive antibodies; eGFR, estimated glomerular filtration rate; FCXM, flow cytometry crossmatch; KT, kidney transplant; MFI, mean fluorescence intensity; MPA, mycophenolate acid; mTORi, mammalian target of rapamycin inhibitor; pfDSA, preformed donor-specific antibody; HLA, human leukocyte antigen; TAC, tacrolimus.

Overall patient survival was 94% at 1 year and 74% at 5 years, with no significant differences between sexes (77% in men vs. 73% in women; log-rank *P* = .53). Causes of death included infectious diseases (49%), cardiovascular diseases (31%), and neoplasms (10%). Notably, this study included patients transplanted during the COVID-19 pandemic, with 13% of deaths attributable to SARS-CoV-2 infection.

### Acute rejection and graft survival

Death-censored graft survival was 97% at 1 year and 82% at 5 years. Within the first year, 75 recipients (17%) experienced acute rejection, of which 51 episodes (68%) were AMR or mixed rejection. Five-year graft survival was significantly lower in recipients with AMR (38%), compared with those with TCMR (71%) or no rejection (88%) (log-rank *P* < .001; Fig. 2). Beyond the first year, 22 recipients (5.0% of the overall cohort) without previous first-year AMR developed AMR between years 1 and 5. Among these patients, three (13.6%) experienced graft failure within 5 years.

**Figure 2:**
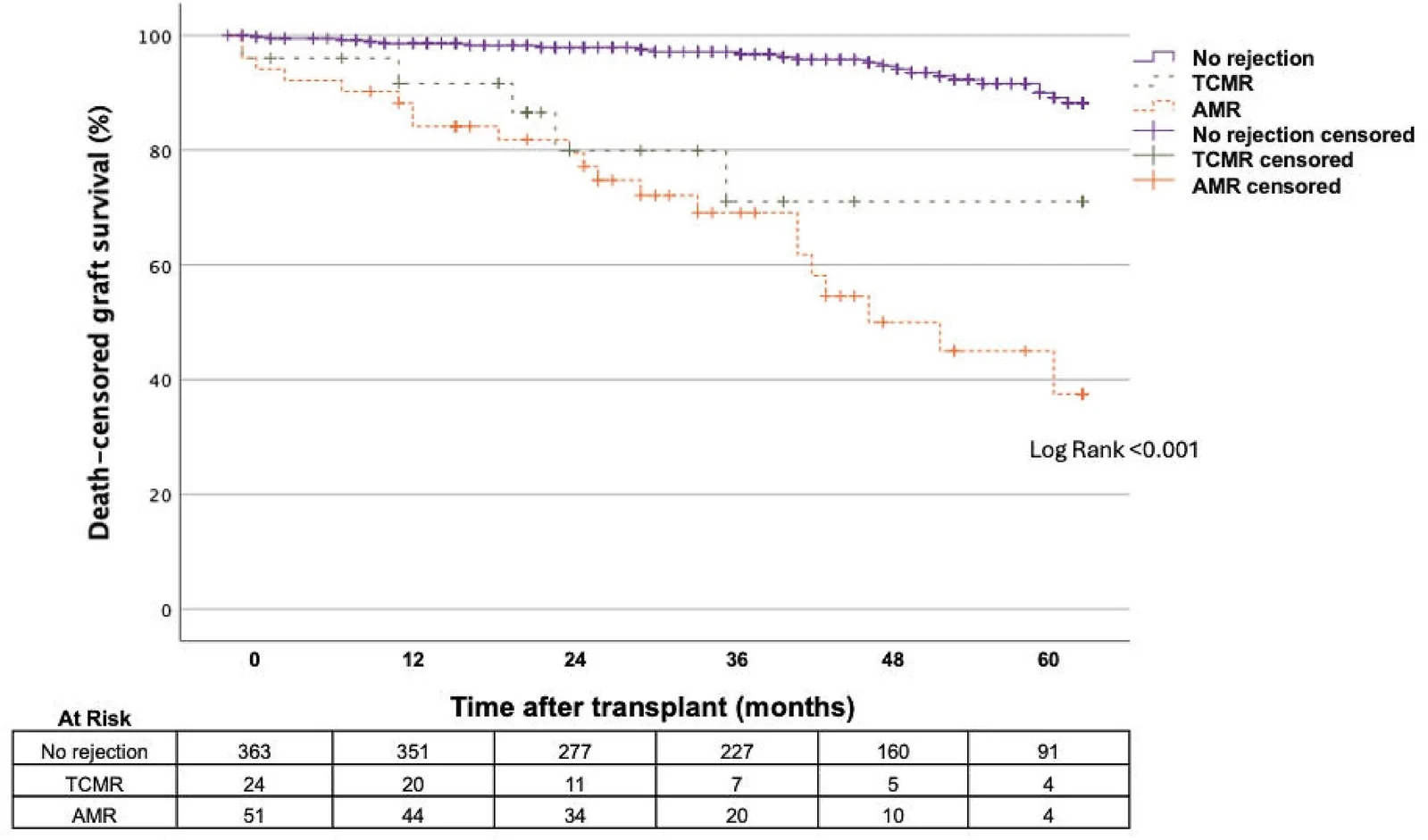
Death-censored graft survival according to rejection type during the first post-transplant year. Survival according to rejection phenotype. Only rejection episodes occurring within the first post-transplant year were considered, and graft survival was assessed over a follow-up of up to 5 years. Five-year death-censored graft survival was 88% in recipients without rejection, 71% in those with TCMR, and 38% in those with AMR (log-rank *P* < .001).

In univariable analysis, 1-year AMR was strongly associated with graft failure (HR 6.3; 95% CI 2.9–13.5). Diabetes and delayed graft function were also associated with graft loss, whereas recipient sex and age were not (Table S1).

In multivariable analysis adjusted for diabetes and delayed graft function, 1-year AMR remained the strongest predictor of graft loss (HR 8.3; 95% CI 4.4–15.7). Diabetes was independently associated with graft failure (HR 2.6; 95% CI 1.4–4.9), whereas delayed graft function showed a borderline association. Recipient sex was not independently associated with 5-year graft loss (HR 1.1; 95% CI 0.6–2.0). Sex-stratified multivariable analyses showed that 1-year AMR remained the strongest predictor of graft failure in both men (HR 4.3; 95% CI 1.8–10.4) and women (HR 17.6; 95% CI 7.2–43.5), with a larger effect estimate observed among women. Diabetes was independently associated with graft loss in both sexes, whereas delayed graft function was associated with graft failure only among men (Table S2).

### Sex differences in AMR risk

Within the first-year post-transplant, 51 recipients (11.6%) developed AMR, with a higher incidence of AMR in men than in women (16% vs. 9%; *P* = .013). Kaplan–Meier 1-year AMR-free survival was 83% in men and 91% in women (log-rank *P* = .011; Fig. 3), with early divergence within the first 60 days. AMR was consistently associated with younger recipient age (<50 years) in both sexes (men: 59% vs. 33%, *P* = .01; women: 55% vs. 27%, *P* = .01).

**Figure 3:**
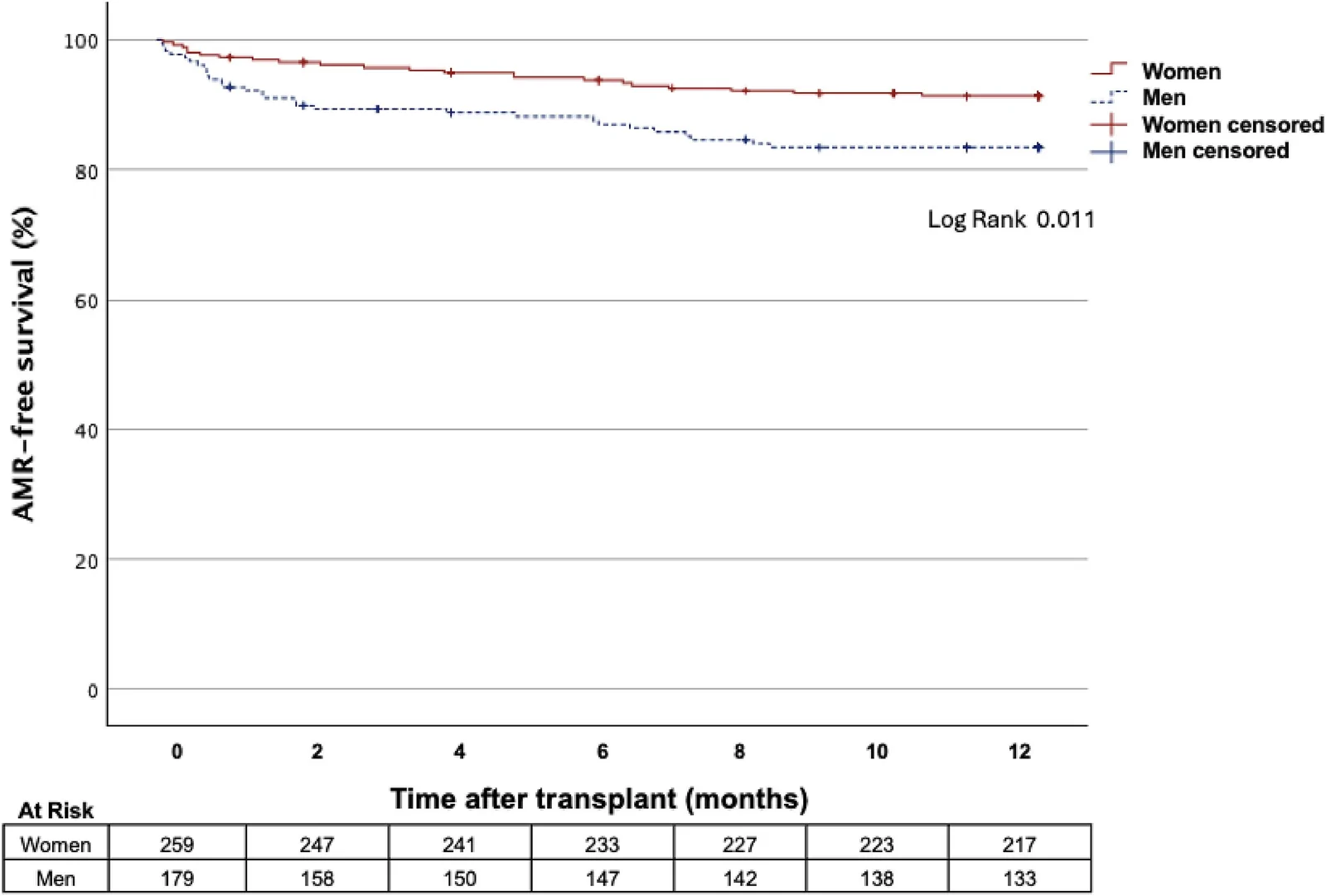
AMR-free survival stratified by recipient sex. Kaplan–Meier curves showing AMR-free survival within the first year after transplantation in women and men. Men displayed a significantly higher incidence of AMR, with early separation of survival curves (log-rank *P* = .011). One-year AMR-free survival was 91% in women and 83% in men. Numbers at risk are provided below the figure.

In univariable Cox regression, male sex (HR 2.1; 95% CI, 1.2–3.8), recipient age <50 years, higher DSA MFI (≥4100) and HLA-DQB1 eplet mismatch >4 (with cut-offs derived from ROC analysis) were associated with 1-year AMR (Table 2).

**Table 2:** Univariate predictors of 1-year AMR in the overall cohort.

	Univariate HR (95% CI)
Recipient sex (men)	2.1 (1.2–3.8)
Recipient age < 50	3.2 (1.7–5.7)
Donor sex (women)	1.4 (0.8–2.4)
# Previous KT	1.8 (1.1–3.0)
cPRA (%)	1.1 (1.0–1.3)
FCXM (positive)	2.5 (1.0–5.9)
pfDSA at transplant	3.8 (2.1–6.9)
pfDSA MFI <4100 vs. none	1.7 (0.8–3.5)
pfDSA MFI ≥4100 vs. none	5.1 (2.7–9.6)
Class II pfDSA positive	2.5 (1.3–5.1)
HLA-DQB1 # MM	1.6 (1.0–2.6)
All HLA Class II eplet MM	1.0 (1.0–1.1)
Verified HLA Class II eplet MM	1.1 (1.0–1.2)
All HLA-DQB1 eplet MM >4	2.3 (1.2–4.2)
Verified HLA-DQB1 eplet MM >2	2.9 (1.6–5.5)
Verified HLA-DQA1 eplet MM >1	1.6 (0.9–3.0)

Abbreviations: CI, confidence interval; cPRA, calculated panel reactive antibody; HLA, human leukocyte antigen; HR, hazard ratios; FCXM, flow cytometry crossmatch; KT, kidney transplant; MFI, mean fluorescence intensity; MM, mismatches; pfDSA, preformed donor-specific antibody.

To determine whether the association between sex and AMR was independent of baseline immunologic risk, we constructed pre-specified sequential Cox models (Table 3). Because previous kidney transplantation was substantially more frequent among male recipients and represents an important determinant of alloimmune memory, it was retained as an a priori covariate in all models regardless of statistical significance to account for its potential confounding effect.

**Table 3:** Sequential multivariable Cox models for 1-year AMR. Hazard ratios with 95% confidence intervals are shown.

Variable	Model 1 Clinical	Model 2	Model 3	Model 4 primary	Model 5^a^
Male sex	2.1 (1.1–3.8)	1.9 (1.1–3.2)	2.1 (1.1–3.8)	1.9 (1.0–3.5)	1.7 (0.9–3.0)
Age < 50 years	2.9 (1.6–5.0)	2.6 (1.5–4.5)	2.5 (1.5–4.5)	2.7 (1.5–4.8)	3.0 (1.6–5.5)
Previous kidney transplant	1.4 (0.7–2.8)	1.6 (0.8–3.3)	1.4 (0.7–2.9)	1.3 (0.7–2.6)	1.3 (0.7–2.3)
cPRA (per unit)	—	1.1 (1.0–1.2)	—	—	—
pfDSA at transplant	—	—	3.0 (1.7–5.2)	—	—
DSA MFI ≥4100	—	—	—	5.1 (2.7–9.6)	4.1 (2.0–8.2)
DSA MFI <4100	—	—	—	1.8 (0.9–3.8)	1.7 (0.8–3.8)
HLA-DQB1 eplet MM (>4)	—	—	—	2.3 (1.3–4.2)	2.7 (1.4–5.1)
FCXM positive	—	—	—	—	1.6 (0.7–3.8)

Note: Model 1 includes recipient sex, age and previous kidney transplantation. Model 2 adds global sensitization (cPRA). Model 3 incorporates presence of preformed DSA. Model 4 (primary model) includes DSA strength (MFI categories) and HLA-DQB1 eplet mismatch. Model 5 represents sensitivity analysis including FCXM.

Abbreviations: CI, confidence interval; HLA, human leukocyte antigen; cPRA, calculated panel reactive antibody; FCXM, flow cytometry crossmatch; MFI, mean fluorescence intensity; MM, mismatches; pfDSA, preformed donor-specific antibody.

^a^Model 5 restricted to recipients with available FCXM data (*n* = 374).

In the initial clinical model including recipient sex and age category, both male sex (HR 2.1; 95% CI, 1.1–3.8) and recipient age <50 years (HR 2.9; 95% CI, 1.6–5.0) were independently associated with AMR, whereas previous kidney transplantation was not (HR 1.4; 95% CI, 0.7–2.8). Addition of cPRA did not substantially attenuate these estimates, as cPRA was not independently associated with rejection.

Male sex remained independently associated with AMR despite incorporation of the strong predictor pfDSA to the model. Given the established importance of antibody intensity, pfDSA presence was replaced by a categorical variable reflecting DSA strength. High-strength antibodies (MFI ≥4100) were strongly associated with AMR (HR 5.1; 95% CI, 2.7–9.6), whereas lower-strength DSA were not. Similarly, HLA-DQB1 eplet mismatch >4 was independently associated with AMR (HR 2.3; 95% CI, 1.3–4.2). In this adjusted model, male sex continued to show a modest independent association (HR 1.9; 95% CI, 1.0–3.5), along with recipient age <50 years (HR 2.7; 95% CI, 1.5–4.8).

In sensitivity analyses restricted to recipients with available FCXM data, FCXM positivity was not independently associated with AMR (HR 1.6; 95% CI, 0.7–3.8). Inclusion of FCXM modestly attenuated the association between male sex and AMR (HR 1.7; 95% CI, 0.9–3.0), which was no longer statistically significant, whereas the effect estimates for DSA strength and HLA-DQB1 eplet mismatch were minimally affected. These findings support the independent contribution of antibody strength and HLA-DQB1 mismatch to early rejection risk.

As an additional sensitivity analysis addressing baseline differences between sexes, stabilized IPTW was applied. Weighting achieved adequate balance across all prespecified baseline covariates (all absolute SMDs < 0.10). Most notably, the marked imbalance in previous kidney transplantation decreased from an SMD of 0.86 before weighting (91.1% in men vs. 57.1% in women) to 0.018 after weighting (71.8% vs. 71.0%). In the IPTW-weighted Cox model, male recipient sex remained associated with a higher risk of 1-year AMR (HR 2.09; 95% CI, 1.10–3.97; *P* = .023). The proportional hazards assumption was not violated (*P* = .252) (Table S3).

We next evaluated whether the association between recipient sex and AMR differed according to retransplantation status. No statistically significant interaction between recipient sex and previous kidney transplantation was observed (interaction HR 2.91; *P* = .21).

Finally, donor–recipient sex combination was incorporated into the fully adjusted model as an additional sensitivity analysis. Donor–recipient sex combination was not independently associated with 1-year AMR (overall Wald test, *P* = .105), and its inclusion did not materially modify the associations observed for recipient sex or the other covariates (Table S4).

### Sex-stratified determinants of AMR

Immunologic risk profiles at transplantation differed by sex and by AMR status (Table 1). Among men, FCXM positivity (24% vs. 5%; *P* < .001) and the presence of pfDSA at the day of transplantation (66% vs. 20%; *P* < .001) were more frequent in those who developed AMR. These pfDSA were most often of class II (24% vs. 9%; *P* = .02) and had higher MFI (≥4100) (68% vs. 27%; *P* = .01) compared with men who did not develop AMR. In women, the pattern differed. FCXM positivity was observed among women without AMR (*n* = 12; 6%) but in none of those who developed AMR (0%; *P* = .26). pf DSA at transplantation were more frequent in women with AMR than without AMR (36% vs. 25%) but failed to reach statistical significance (*P* = .24). In contrast, immunogenetic compatibility metrics were more strongly associated with AMR among women. Women with AMR had higher HLA-DQB1 antigen mismatch (*P* = .03) and class II eplet mismatches (total eplets, *P* = .04; verified eplets, *P* = .02). Threshold-based DQB1 eplet measures showed particularly strong enrichment: Total HLA-DQB1 eplet mismatches >4 were present in 77% of women with AMR vs. 45% without AMR (*P* = .003), and verified HLA-DQB1 eplet mismatch >2 was present in 73% of women with AMR vs. 31% without AMR (*P* < .001).

Given differences in baseline immunologic profiles between sexes and persistence of sex as an independent predictor, exploratory sex-stratified Cox models were performed (Table 4). Among men, antibody strength was the dominant determinant of rejection. High-strength DSA (MFI ≥ 4100) was associated with an increased hazard of AMR (HR 3.1; 95% CI, 1.9–5.2). In contrast, HLA-DQB1 eplet mismatch was not independently associated with AMR in men (HR 1.7; 95% CI, 0.8–3.5). Among women, the pattern differed. Antibody intensity was not independently associated with rejection, whereas HLA-DQB1 eplet mismatch above threshold was strongly associated with AMR (HR 4.2; 95% CI, 1.6–11.5).

**Table 4:** Sex-stratified multivariable cox models for 1-year AMR.

Variable	Men (*n* 179; 29 events) HR (95% CI)	Women (*n* 259; 22 events) HR (95% CI)
Age < 50 years	2.0 (1.0–4.2)	3.4 (1.4–7.8)
pfDSA MFI (overall test)		
DSA MFI <4100	0.3 (0.2–0.6)	0.8 (0.4–1.4)
DSA MFI ≥4100	3.1 (1.9–5.2)	1.6 (0.7–3.3)
HLA-DQB1 eplet MM (>4)	1.7 (0.8–3.5)	4.2 (1.6–11.5)

Abbreviations: CI, confidence interval; HLA, human leukocyte antigen; HR, hazard ratios; MFI, mean fluorescence intensity; MM, mismatches; pfDSA, preformed donor-specific antibody.

Formal interaction testing was performed to assess whether the effect of antibody strength or immunogenetic mismatch differed by sex. The interaction between sex and DQB1 eplet mismatch did not reach statistical significance (*P* = .11), although point estimates suggested attenuation of the DQB effect among men compared with women. Similarly, the interaction between sex and antibody intensity demonstrated a non-significant trend (*P* = .07).

These findings suggest potential sex-related differences in the relative contribution of antibody burden and immunogenetic compatibility to early rejection risk; however, limited event numbers reduce power to detect definitive effect modification.

### Sex-specific differences in causes of graft failure

Among the 49 graft losses observed within 5 years, the distribution of causes differed significantly between sexes (Fisher–Freeman–Halton exact test, *P* = .010). Immunological causes accounted for a greater proportion of graft failures in men than in women (87.5% vs. 56.0%), whereas infection-related graft loss occurred exclusively in women (28.0% vs. 0%), mainly due to recurrent pyelonephritis (24.0% of female graft losses). Other causes of graft loss were similarly distributed between sexes (Table S5).

## DISCUSSION

In this multicenter cohort of highly sensitized recipients with cPRA ≥90%, we observed that men experienced a higher crude incidence of 1-year AMR compared with women. In sequential multivariable models incorporating recipient age, pfDSAs, antibody strength, and HLA-DQB1 eplet mismatch, male sex remained associated with early AMR, although the magnitude of the effect was moderate and estimates were sensitive to model specification. This association was further supported by the IPTW sensitivity analysis, which achieved adequate balance in measured baseline characteristics, including the marked difference in previous kidney transplantation. These findings suggest that within an extreme immunologic risk context, biological sex may be associated with early rejection risk beyond conventional measures of antibody burden and antigen-level compatibility. Nonetheless, given the observational design and limited number of events, residual confounding cannot be excluded.

The role of sex in kidney transplant outcomes remains incompletely described. In general transplant populations, female sex has often been associated with higher sensitization rates, largely driven by pregnancy-related HLA exposure and reduced access to compatible donors [20]. However, after adjustment for immunologic risk markers, sex has not consistently emerged as an independent determinant of acute rejection or graft loss in registry-based analyses. Systematic reviews and meta-analyses emphasize heterogeneity across cohorts and study designs, and overall certainty of evidence regarding sex-based outcome differences remains low [15]. Our study contributes to this field by examining a carefully phenotyped, uniformly high-risk population in which antibody burden and molecular compatibility were rigorously characterized.

A notable finding was that antibody intensity and immunogenetic mismatch appeared to represent partially independent axes of AMR risk. High-strength DSA (MFI ≥ 4100) was strongly associated with early rejection in the overall cohort, consistent with prior evidence that antibody strength is a major determinant of antibody-mediated injury [3, 7]. Simultaneously, HLA-DQB1 eplet mismatch above predefined thresholds was independently associated with AMR, reinforcing the concept that fine-scale molecular incompatibility conveys additional prognostic information beyond conventional antigen-level mismatch [8, 21].

Exploratory sex-stratified analyses suggested that these immunologic dimensions may not contribute equally across sexes. In men, antibody strength appeared to be the principal correlate of rejection, whereas DQB1 eplet mismatch did not independently predict AMR. Among women, the opposite pattern was observed: DQB1 eplet mismatch demonstrated stronger associations, whereas antibody intensity was not independently predictive. Although biologically intriguing, these subgroup findings must be interpreted cautiously. Formal interaction testing did not consistently demonstrate statistically robust effect modification, and the limited number of AMR events reduces statistical power. Therefore, we cannot conclude that the immunologic architecture of rejection differs by sex; rather, our data raise the hypothesis that antibody burden and molecular mismatch may interact differently across sexes in highly sensitized recipients.

The observation that male sex remained independently associated with AMR after adjustment for cPRA, DSA intensity, and HLA-DQB1 eplet mismatch suggests that conventional quantitative metrics may not fully capture alloimmune risk in highly sensitized recipients. Emerging evidence indicates that qualitative features of the humoral response, including complement-binding capacity (e.g. C1q positivity) [22], immunoglobulin G (IgG) subclass distribution (particularly IgG1 and IgG3 predominance) [23, 24], antibody Fc glycosylation patterns, and downstream Fc-effector engagement [25], substantially influence antibody pathogenicity beyond MFI thresholds. Complement-binding DSA and IgG3-enriched profiles have been associated with more severe AMR phenotypes and inferior graft outcomes, independent of antibody strength. Moreover, proinflammatory Fc glycosylation signatures, including hypogalactosylation, have been associated with enhanced effector function and antibody-mediated injury. Given known sex-based differences in humoral immune regulation and hormone-dependent modulation of IgG Fc glycosylation, it is biologically plausible that specific molecular mismatch patterns may have differential immunogenic consequences across sexes [26]. Prospective studies incorporating functional antibody profiling alongside molecular HLA matching are needed to clarify these mechanisms and guide precision immunosuppression in highly sensitized patients.

Beyond determinants of early rejection, AMR had a profound adverse impact on long-term graft survival. One-year AMR was the strongest independent predictor of 5-year death-censored graft failure, consistent with prior reports linking early antibody-mediated injury to chronic microvascular damage and late graft loss [7, 27]. Diabetes and delayed graft function also contributed to graft failure. In contrast, recipient sex was not independently associated with 5-year graft survival after accounting for AMR and clinical variables. Thus, in this cohort, sex did not directly influence long-term graft survival independent of rejection events. These findings suggest that any potential influence of sex on long-term graft outcome may be more closely related to differences in early AMR than to an independent effect of sex on graft survival. However, late AMR events were not incorporated as time-dependent covariates; therefore, the potential contribution of subsequent immunological events to 5-year graft outcomes cannot be fully excluded.

Sex-stratified survival analyses suggested that the magnitude of AMR-associated graft failure may differ between men and women, with larger point estimates observed in women. Sex-based differences in humoral immune responsiveness provide biological plausibility for effect modification [28]. However, these findings should be interpreted with caution due to the modest number of graft failure events (*n* = 49) and may not reflect true biological divergence.

We also observed sex-related differences in attributed causes of graft failure and infectious complications. Immunologic causes predominated overall and were more common among men, whereas women had a higher proportion of infection-related graft loss, primarily from recurrent pyelonephritis, despite similar rates of major viral replication. Although infections represented a substantial cause of mortality, they were considered post-transplant events rather than baseline confounders in the death-censored graft-survival analyses. Prior studies have reported higher rates of urinary tract infections among women after transplantation [29], indicating that post-transplant clinical trajectories may differ by sex. However, our study could not distinguish between biological susceptibility, cumulative immunosuppressive exposure, pharmacokinetic variability, medication adherence, or contextual factors that may influence infection risk and graft outcomes [15, 30]. Accordingly, these observations should be interpreted as hypothesis-generating rather than evidence of sex-specific causal mechanisms.

This study has several limitations. Kidney biopsy diagnoses were based on the original pathology reports and were not centrally re-reviewed or retrospectively reclassified. As the Banff classification has evolved during the study period, particularly regarding the diagnosis of AMR, some biopsies initially interpreted as non-rejection might be classified differently using current diagnostic criteria. Although this may have led to a modest underestimation of rejection events, particularly cases that under more recent Banff classifications might be categorized as probable AMR or microvascular inflammation (MVI)-positive phenotypes, it is more likely to have affected borderline or previously unclassified biopsies than those fulfilling established diagnostic criteria for AMR. Therefore, this is unlikely to have materially influenced the principal findings of the study. An additional limitation is that protocol biopsy practices were not fully standardized across centers, and information on biopsy indications and the proportion of subclinical versus clinically overt AMR was not systematically available. Therefore, differences in surveillance practices may have influenced the detection of subclinical rejection, although all participating centers followed routine clinical practice within the regional transplant network.

In addition, the relatively limited number of AMR events constrained the complexity of the multivariable models and may have reduced the precision of the fully adjusted estimates.

Finally, standardized measures of medication adherence and longitudinal information on post-transplant immunosuppressive modifications, serial DSA monitoring, and biopsy reclassification were not systematically available. Detailed data on severe infections preceding graft failure and temporary reductions in immunosuppression were also not systematically collected; therefore, the potential contribution of an infection–immunosuppression reduction–rejection pathway to graft loss could not be assessed. Because nonadherence may influence rejection and graft outcomes and may differ between sexes, its potential contribution to the observed associations could not be evaluated and cannot be excluded. These factors should be addressed in future prospective studies.

Despite these limitations, this study includes one of the largest multicenter cohorts of highly sensitized kidney transplant recipients with comprehensive immunological characterization. The sequential adjustment for conventional and molecular immunological risk factors, complemented by the IPTW sensitivity analysis, provide a robust framework for evaluating the independent association between recipient sex and early AMR.

In summary, in highly sensitized kidney transplant recipients, early AMR remains the dominant determinant of long-term graft survival. Male sex was associated with early rejection in adjusted analyses but did not independently predict 5-year graft loss. These findings reinforce the multidimensional nature of humoral risk, highlighting the complementary contributions of antibody strength and molecular HLA compatibility. Although our data raise the possibility that the relative contribution of these factors may differ by sex, larger cohorts integrating functional antibody characterization and detailed donor–recipient sex data will be necessary to determine whether sex truly modifies alloimmune biology or reflects unmeasured residual differences beyond current immunologic metrics.

## Supplementary Material

sfag367_Supplemental_File

## Data Availability

The data underlying this article will be shared on reasonable request to the corresponding author.
