# Supplementary material for: Sex-based differences in antibody-mediated rejection among highly sensitized kidney transplant recipients
Source: Clin Kidney J. 2026 Oct 19;19(11):sfag367. doi: 10.1093/ckj/sfag367 (PMC13748982; doi:10.1093/ckj/sfag367)
Supplement: Supplementary file 1 [file sfag367_supplemental_file.docx]

**Table S1.** Univariable Predictors of Five-Year Death-Censored Graft Failure.

|  | HR (95% CI) |
| --- | --- |
| One-year AMR | 6.3 (2.9-13.5) |
| Recipient sex (men) | 1.4 (0.8-2.6) |
| Recipient age <50 years | 1.3 (0.6-2.7) |
| Diabetes | 2.0 (1.0-6.2) |
| Delayed graft function | 2.3 (1.2-4.0) |
| Verified HLA-DQB1 eplet MM >2 | 1.9 (1.0-3.9) |

CI, confidence interval; Hazard ratios derived from univariable Cox proportional hazards models. Outcome: five-year death-censored graft failure. Total events: n. 49.

AMR, antibody-mediated rejection; HLA, human leukocyte antigen; MM, mismatches.

**Table S2**. Sex-Stratified Multivariable Cox Models for Five-Year Death-Censored Graft Failure

| Variable | Men  (n.179; 24 events)  HR (95% CI) | Women  (n. 259; 25 events)  HR (95% CI) |
| --- | --- | --- |
| One-year AMR | 4.3 (1.8-10.4) | 17.6 (7.2-43.5) |
| Diabetes | 3.0 (1.3-7.2) | 3.5 (1.3-9.6) |
| Delayed graft function | 2.9 (1.2-7.0) | 0.9 (0.3-2.6) |

CI, confidence interval; Hazard ratios derived from sex-stratified multivariable Cox proportional hazards models adjusted for diabetes and delayed graft function. Outcome: five-year death-censored graft failure. Subgroup analyses are exploratory and based on limited event numbers (total events: 49) and should be interpreted cautiously.

**Table S3.** Covariate balance and association between recipient sex and one-year AMR after inverse probability of treatment weighting.

A. Covariate balance

| Baseline covariate | Women unweighted | Men unweighted | Absolute SMD before IPTW | Women weighted | Men weighted | Absolute SMD after IPTW |
| --- | --- | --- | --- | --- | --- | --- |
| Age (y), mean (SD) | 55 (12) | 54 (13) | 0.09 | 55 (12) | 55 (13) | 0.03 |
| Previous KT, n. (%) | 148 (57) | 163 (91) | 0.86 | 71% | 72% | 0.02 |
| cPRA (%), mean (SD) | 97 (3) | 97 (3) | 0.08 | 97 (3) | 97 (3) | 0.02 |
| Transfusions before KT, n. (%) | 119 (46) | 82 (46) | <0.01 | 46% | 48% | 0.04 |
| pfDSA at transplant, n. (%) | 67 (26) | 49 (27) | 0.03 | 27% | 30% | 0.05 |
| HLA-DQB1 eplet MM, mean (DS) | 5 (6) | 5 (5) | <0.01 | 6 (6) | 5 (5) | 0.03 |

B. IPTW-weighted Cox model for one-year AMR

| Variable | HR (95% CI) | *p* |
| --- | --- | --- |
| Male recipient | 2.1 (1.1–4.0) | 0.02 |

AMR, antibody-mediated rejection; cPRA, calculated panel reactive antibody; HLA, human leukocyte antigen; HR, hazard ratio; IPTW, inverse probability of treatment weighting; KT, kidney transplant; MM, mismatch; pfDSA, preformed donor-specific antibody; SMD, standardized mean difference. Absolute SMD <0.10 was considered indicative of adequate balance.

**Table S4.** Multivariable model including donor–recipient sex combination for prediction of one-year AMR

| Variable | Adjusted HR (95% CI) |
| --- | --- |
| Donor–recipient sex combination (overall test) | — |
| Female recipient – Female donor | Reference |
| Male recipient – Male donor | 1.1 (0.4–2.7) |
| Female recipient – Male donor | 0.4 (0.1–1.0) |
| Male recipient – Female donor | 0.9 (0.3–2.5) |
| Previous kidney transplantation | 1.2 (0.5–2.9) |
| Positive FCXM | 1.7 (0.6–5.1) |
| Pretransplant DSA MFI ≥4100 | 6.7 (2.9–15.2) |
| HLA-DQB1 eplet mismatch (>4) | 2.8 (1.4–5.7) |

AMR, antibody-mediated rejection; DSA, donor-specific antibody; FCXM, flow cytometric crossmatch; HLA, human leukocyte antigen; MFI, mean fluorescence intensity.

**Table S5.** Causes of five-year kidney graft failure stratified by sex.

|  | Total  n= 49 | Men  n= 24 | Women  n= 25 |
| --- | --- | --- | --- |
| Immunological n. (%) | 35 (71.4) | 21 (87.5) | 14 (56.0) |
| Chronic graft dysfunction without histological study n. (%) | 11 (22.4) | 8 (33.3) | 3 (12.0) |
| cAMR n. (%) | 11 (22.4) | 4 (16.7) | 7 (28.0) |
| aAMR n. (%) | 10 (20.4) | 7 (29.2) | 3 (12.0) |
| aTCMR n. (%) | 3 (6.1) | 2 (8.3) | 1 (4.0) |
| Infection n. (%) | 7 (14.3) | 0 (0.0) | 7 (28.0) |
| Pyelonephritis n. (%) | 6 (12.2) | 0 (0.0) | 6 (24.0) |
| BK virus nephropathy n. (%) | 1 (2.0) | 0 (0.0) | 1 (4.0) |
| Glomerular disease recurrence n. (%) | 1 (2.0) | 0 (0.0) | 1 (4.0) |
| Others or undetermined causes n. (%) | 6 (12.2) | 3 (12.5) | 3 (12.0) |

Pearson Chi-Square: 9.38, df = 3, p = 0.025 (Exact p = 0.014), Likelihood Ratio: 12.48, df = 3, p = 0.006 (Exact p = 0.010). Fisher-Freeman-Halton Exact Test: 9.68, p = 0.010

cAMR, chronic antibody-mediated rejection; aAMR, acute antibody-mediated rejection; aTCMR, acute T cell-mediated rejection.
